# Spatial networks and the spread of COVID-19: results and policy implications from Germany

**DOI:** 10.1007/s10037-023-00185-6

**Published:** 2023-05-15

**Authors:** Matthias Flückiger, Markus Ludwig

**Affiliations:** 1 University of York, York, United Kingdom; 2 CESifo, Munich, Germany; 3 Technische Universität Braunschweig, Braunschweig, Germany

**Keywords:** I12, I15, I18, H51, C23, COVID-19, Spatial networks, Public policy

## Abstract

Spatial networks are known to be informative about the spatiotemporal transmission dynamics of COVID-19. Using district-level panel data from Germany that cover the first 22 weeks of 2020, we show that mobility, commuter and social networks all predict the spatiotemporal propagation of the epidemic. The main innovation of our approach is that it incorporates the whole network and updated information on case numbers across districts over time. We find that when disease incidence increases in network neighbouring regions, case numbers in the home district surge one week later. The magnitude of these network transmission effects is comparable to within-district transmission, illustrating the importance of networks as drivers of local disease dynamics. After the introduction of containment policies in mid-March, network transmission intensity drops substantially. Our analysis suggests that this reduction is primarily due to a change in quality—not quantity—of interregional movements. This implies that blanket mobility restrictions are not a prerequisite for containing the interregional spread of COVID-19.

## Introduction

COVID-19 has spread rapidly across the world, with 188 countries reporting at least one COVID-19 case.^1^ In many Western countries, community transmission is pervasive. Because regions, particularly within the same nation, are interlinked, this implies that local COVID-19 transmission dynamics not only depend on community transmission rates, but also on disease dynamics in other regions. Local flare-ups can spill over into other regions. Understanding patterns and extent of such spillovers becomes particularly relevant now, as countries are beginning to lift nation-wide lockdowns and move to more localised responses. This requires identifying relevant spatial networks, i.e., networks that help predict the spatiotemporal diffusion of the disease and investigating their effect under different containment policy regimes. Contributing to this understanding is the aim of this paper.

In this paper, we focus on Germany and analyse the influence of three different types of spatial networks on the spread of COVID-19 across districts over time and containment strategies. The three networks are: the mobility network, the commuter network, and the social network. The strength of connection between two districts within the mobility network is measured via phone-location-tracking-derived movement of individuals, whereas connectivity within the commuter network is based on data from the Federal Employment Agency. The strength of links within the social network is measured by the Social Connectedness Index (Bailey et al 2020), which captures the intensity of Facebook friendships between two districts. It is important to note that the structure of the networks (i.e., the strength of links) is time-invariant and pre-determined, i.e., reflecting typical patterns of connectivity prior to the outbreak of COVID-19.

To investigate if the three networks influence interdistrict transmission dynamics, we compile a dataset on weekly incidence rates (defined as the number of new COVID-19 cases per inhabitant) for each of the 401 German districts. This district-level panel dataset spans the period 1 January 2020–31 May 2020. We link disease incidence in the remaining districts to the home district by averaging COVID-19 incidence rates across all other districts using connectivity within a given network as weight. This measure thus captures the network-proximity-weighted average COVID-19 incidence rate of all other districts. The intuition behind this index is that disease dynamics in other districts are more relevant the closer districts are connected to the home district. Importantly, this measure incorporates the whole network (i.e., all districts) and updated information on case numbers. This is—to the best of our knowledge—novel and allows us to investigate the effect of networks on the spatiotemporal propagation of the disease beyond initial stages.

Using the panel data, we then employ an OLS regression approach to empirically assess if lagged changes in network-proximity-weighted average COVID-19 incidence rate predicts disease incidence in the home district. Our regression setup accounts for district fixed effects as well as state $\times$ week dummies. The latter absorb general (within-state) disease dynamics as well as variation in state-wide containment policies. The former control for differences in district-level characteristics that could influence disease transmission intensity (e.g. population density or distance to initial hotspots). Furthermore, we always include lagged incidence rates observed in the home district to control for within-district transmission dynamics.

The regressions produce two main results. First, the structure of all three networks is predictive of the spatiotemporal diffusion of COVID-19. Case incidence rises in the home district following an increase in the network-proximity-weighted average COVID-19 incidence rate. The size of the point estimators for the network effects are statistically indistinguishable from within-district dynamics, illustrating the importance of taking into account network effects when trying to understand the spatiotemporal spread of the COVID-19 epidemic. Among the three networks, the social network has the strongest effect and contains the most information (as measured by the Bayesian information criterion).

The second main result is that the intensity of transmission within all spatial networks drops substantially (below the critical value of one) after the introduction of containment policies in week 12 (16 March 2020). Using information on daily observed mobility (rather than time-invariant, typical mobility patterns), we show that neither changes in the (relative) pattern of mobility nor changes in the quantity of movement can explain the reduction in network transmission intensity. Though not empirically testable, the natural conjecture then is that qualitative aspects of mobility must have changed. Possible aspects are a change in mode of transport or the adherence to physical distancing guidelines. A main policy implication of our findings is that changing qualitative aspects of mobility are far more important in slowing down the spread of the disease across space than the reduction of mobility itself.

The remainder of this paper is structured as follows: The next section discusses the literature related to our study, which is followed by a brief overview of the COVID-epidemic in Germany during the first 22 weeks of 2020. In Sect. 4, we present the data used in our analysis before outlining the estimation approach in Sect. 5. The results are discussed in Sect. 6, while Sect. 7 offers concluding remarks.

## Literature

Closely related to our paper is the strand of literature that investigates the importance of spatial networks in explaining the diffusion of COVID-19. For Germany, Schlosser et al (2020), Fritz and Kauermann (2022) and Fritz et al (2022) show how network connectedness (mobility and social network) predict the propagation of the disease. Smolyak et al (2021) highlight the importance of human mobility restrictions in slowing COVID-19 diffusion in Italy. For Ahmedabad, India, Patil et al (2021) provide evidence that contact networks and road connectivity patterns correlate with the rate of transmission of COVID-19. Compared to these studies, we go further by highlighting that the quality not the quantity of contacts seem to explain the decline in network transmission after the introduction of containment measures in March 2020. Our work is further differentiated from the previous literature based on the fact that our approach allows us to separate within-location transmission from network diffusion transmission. Additionally, our empirical setup improves existing studies by including unit-of-observation (county) and time fixed effects, which reduce problems related to omitted variables that often makes causal interpretation of network effects difficult. In this aspect, we contribute to a small but growing literature in economics that aims at causally identifying effects of mobility patterns and mobility restrictions on COVID-19 infection rates (Glaeser et al 2022).

Our work further speaks to a small but important literature that is able to map network connectivity at the individual level to investigate its effect on the diffusion of COVID-19. The focus of these studies, however, is generally limited to specific population groups and limited in geographical scope. For example, using the social media network of 93 university students in Spain, Benítez-Andrades et al (2021) document a correlation between network centrality and COVID-19 infection rates. Using 237 cases, Yang (2021) highlight the importance of social networks for the spread of COVID-19 in China.

More broadly, our work fits within the huge literature concerning the importance of mobility, transport infrastructure and, more generally, geographical distance for the spatial diffusion of COVID-19 in the early stages of the epidemic. Since COVID-19 is an infectious disease that spreads through human contact, the literature highlights the imperative importance of transport systems and human mobility for the diffusion of COVID-19 at the international- and subnational level (e.g., Giuliani et al 2020; Harris 2020; Li et al 2020; Kuchler et al 2020; Tian et al 2020; Felbermayr et al 2021). The main focus of the literature lies in describing and predicting the spread rather than in causal inference.

Our findings also speak to the research on effectiveness of COVID-19 containment approaches (Cowling et al 2020; Dehning et al 2020; Hartl et al 2020; Kraemer et al 2020; Flaxman et al 2020; Hsiang et al 2020; Tian et al 2020) and to studies on design of optimal containment policies that incorporate economic trade-offs. The latter have so far focussed on optimal testing strategies as well as policies targeted at specific age groups, health-status groups, sectors, or geographic areas (Acemoglu et al 2020; Chinazzi et al 2020; Eichenbaum et al 2020; Fajgelbaum et al 2020; Glover et al 2020; Rampini 2020). Our results highlight the existence of inter-regional dependencies in disease propagation, suggesting the need for coordination among regions. This does not, however, imply mobility restrictions are necessary to avoid negative (cross-regional) spillovers. Rather, changes in the quality of interpersonal contact—such as adherence to hygiene and social distancing guidelines—may be sufficient for disease containment.

Finally, our work also links into the literature that tries to identify factors that explain varying degrees of compliance with COVID-19-related containment policies, e.g., mobility restrictions. For example, Borgonovi and Andrieu (2020) show that higher levels of social capital are associated with reductions in mobility during the early stages of the epidemic. Similarly, Bargaina and Aminjonovb (2020) find that higher levels of social trust is associated with higher levels of compliance to public health policies.

## Background

**Fig. 1:**
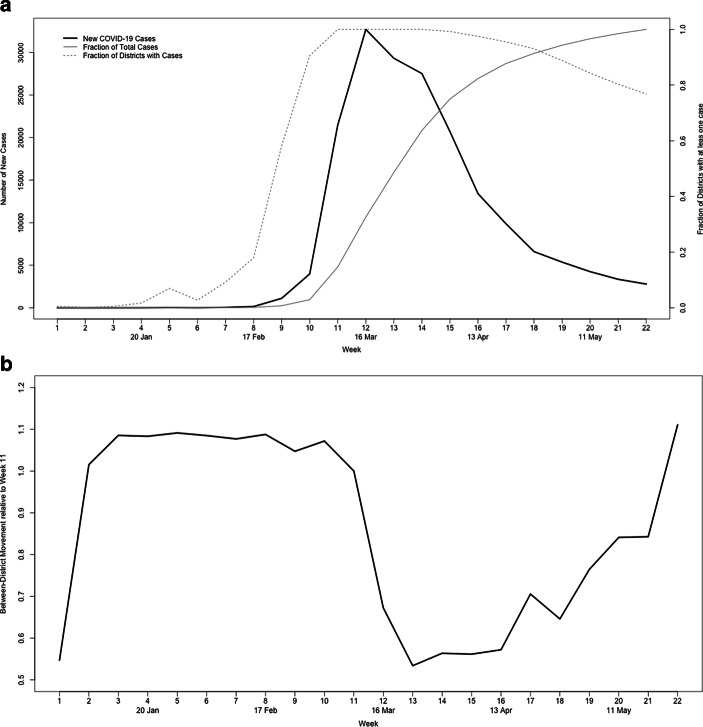
COVID-19 and mobility over time. **a** depicts new weekly COVID-19 infection numbers (data version: 9 June 2020). **b** shows mobility between districts relative to week 11 (data: Teralytics)

Germany reported its first COVID-19 case on 28 January 2020. Retrospectively, the Robert Koch Institute dates the earliest laboratory-confirmed infection back to 1 January 2020.^2^ As depicted in Fig. 1a, case numbers remained relatively low and confined to few districts in January and early February. Starting with the third week of February, however, diffusion across space accelerated dramatically. The fraction of districts reporting COVID-19 cases surged from 9% to 91% in just three weeks. Similarly, the number of new infections rose rapidly; from 152 in week 8 to the peak of 32,693 in week 12.^3^

Reacting to the intensification of transmission, local and national governments first urged people to adhere to hygiene guidelines. Failing to reduce infection numbers, first restrictions were imposed. Effective 9 March, events of more than 1,000 people were banned. One week later (week 12 of 2020), non-essential cross-national border movements were prohibited and schools, childcare facilities as well as many stores closed. Adding to these already existing restrictions, the federal government issued binding guidelines for limiting physical contact with people outside their own household on 22 March 2020 for the whole of Germany.^4^ Similar to other countries all non-essential shops had to close, physical distancing guidelines were enforced by the police, and people were urged to work from home whenever possible.

Overwhelmingly, the German public adhered to the guidelines. Mobile-phone-based mobility data, for example, documents that people limited their movement as a result of government policies. Figure 1b unveils a strong reduction in mobility beginning with week 12, i.e., when schools and borders were closed. By 22 March, the number of trips between districts had dropped to 53% of the pre-epidemic level. For the next four weeks, mobility levels remained low, before they started to rise in week 17, coinciding with public holidays (Ascension Day) and initial relaxations of restrictions.^5^ By the end of the sample period, mobility levels had returned to pre-restriction levels. For the remainder of this paper, we will divide the 22 weeks into two groups: the ‘pre-containment-policy period’ or ‘pre-policy period’ spanning weeks 1–11, and the‘post-policy period’ encompassing weeks 12–22.^6^

## Data

### COVID-19 cases

Data on COVID-19 infections (and COVID-19-related deaths) in Germany across all settings are uniformly recorded nationwide and collated by the Robert Koch Institute (RKI). The spatially most disaggregated version of these data are available at the district level.^7^ In total, there are 401 districts; the median population is 158,000. For each district, we compute the weekly COVID-19 incidence rate—defined as the weekly number of new COVID-19 cases per inhabitant—for the weeks 1–22 (1 January 2020–31 May 2020).^8^

**Fig. 2:**
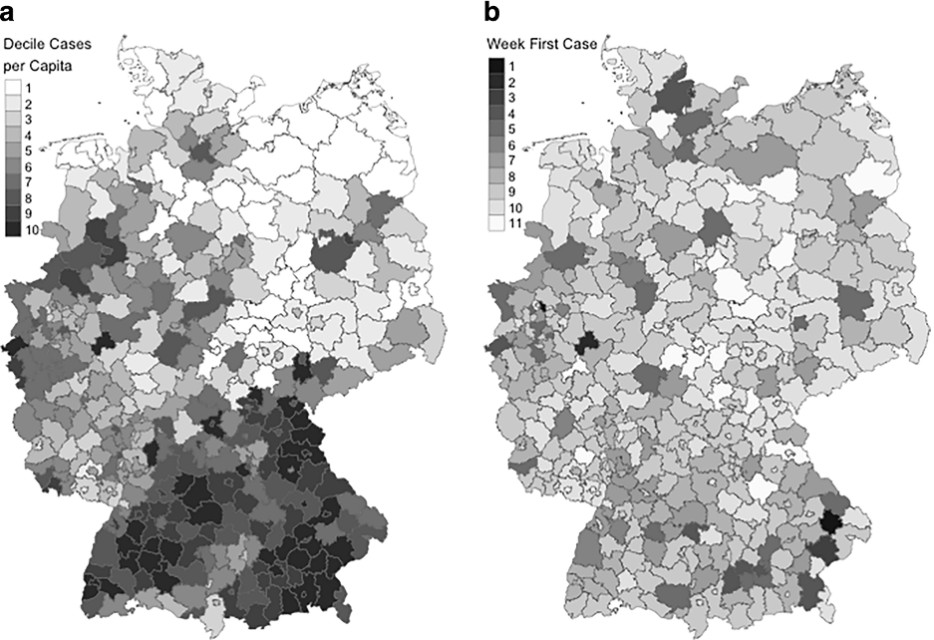
COVID-19 across Districts. Notes: **a** depicts COVID-19 prevalence rates (grouped into deciles). **b** depicts the first week in which a case occurred. Data version: 9 June 2020

To illustrate spatial variation in disease transmission intensity, Fig. 2a depicts the overall COVID-19 prevalence rate (total cases per capita as of week 22) across districts; the darker shadings indicate higher prevalence rates. Clearly discernible are spatially clustered high-transmission-intensity areas in the South and North West. Comparing Fig. 2a and b further suggests that clusters formed around districts into which the virus was introduced very early on. These patterns indicate that spatial networks are important in understanding differences in disease incidence across space and time.

### Spatial networks

To investigate if the structure of spatial networks helps to explain COVID-19 dynamics in the home district, we need a measure that captures variation in the number of secondary cases generated via (close) contact with infected individuals from other districts. This measure should capture the fact that the number of new cases resulting from interaction with an infected individual from district $j$ increases with: (a) the incidence rate in district $j$ in a given week $w$ and (b) the degree of interaction, or flow of people, from district $j$ into $d$. We start the construction of such a measure by first identifying how closely two districts are connected within a given network $N$. To this end, we compute the share of total flows $f_{d,j}$ into district $d$ that originate from district $j$:

1 $$\omega^{N}_{d,j}=\dfrac{f_{d,j}}{\sum_{k=1}^{K}f_{d,k}}.$$

Clearly, connectivity between two districts increases with $\omega^{N}_{d,j}$ and lies between zero and one, irrespective of the level of total inflow into district $d$.^9^ This is equivalent to row-standardising a matrix. Normalisation to one facilitates interpretation. Furthermore, it makes comparison of connectivity across the different types of networks that we look at easier.

The three networks that we focus on are the mobility network, the commuting network, and the social network. Bilateral flows within the mobility network are measured by the total number of individuals that moved between two districts during the year 2019. Movements are captured via phone location tracking and are provided by Teralytics.^10^^,^^11^ The flows between two districts within the commuter network is equal to the total number of individuals that resided in a given district $d$ and worked in another district $j$ (or vice versa) in 2019. District-pair level data on commuter patterns are published by the Federal Employment Agency.^12^ Finally, bilateral connectivity within the social network is measured by the Social Connectedness Index (SCI), developed and described in detail in Bailey et al (2020).^13^ The SCI captures the relative probability of a Facebook friendship link between a given Facebook user in district $d$ and a given user in district $j$. Thus social interaction between districts increases in the SCI.^14^ It is important to note that the structure of all three networks is predetermined, i.e., reflecting typical (average) patterns of interaction prior to 2020. An immediate concern is that the structures change during the COVID-19 epidemic. We address this issue in Sect. 6.

**Fig. 3:**
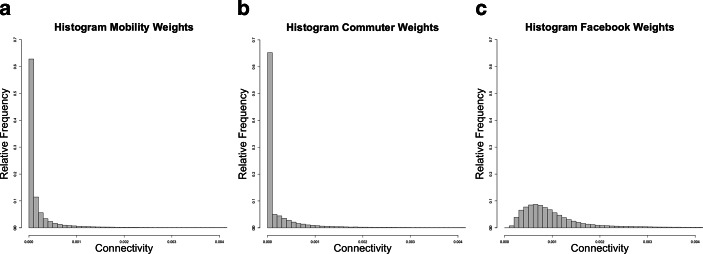
Network connectivity densities. Notes: The figure shows the density plots of the network connectivities for: **a** the mobility network. **b** the commuter network. **c** the social media network (Facebook)

To gain an insight into the degree to which in the structure the three networks differs, Fig. 3 depicts the densities of the connectivities (i.e., $\omega^{N}_{d,j}$) separately for each network. It is clearly visible that the mobility- and commuter network are very similar in their structure. For both networks, the density plots are skewed heavily to the left, indicating that the for a typical county connectivity is very high to relatively few counties while connectivity is low to the vast majority of remaining counties.On the other hand, the density plot for the social media connectivity indicates a more equal distribution, implying that the reach of the social media network is much broader (i.e. less spatially concentrated).

Naturally, connectivities within the three types of networks are correlated. For example, districts linked closely within the commuter network will also maintain more social connections. Similarly, commuter flows are correlated with the total number of people moving from one district to another. The similarity in the structure of the networks becomes evident when looking at the correlation between the bilateral connectivities of the different networks (see Correlation Table 4). Bivariate correlation between bilateral connectivity—defined according to equation (1)—within the mobility and commuting network is 0.97. The association between the mobility network and social network is somewhat weaker, but still high with a value of 0.86.^15^

It is important to note that we will not be able to isolate the effects of an individual network on cross-district COVID-19 transmission dynamics. Rather, our aim is to compare the effect and information content of the three networks with respect to the spatiotemporal transmission of the disease.

Using the information on connectivity $\omega^{N}_{d,j}$ between district-pairs, we can compute the network-proximity-weighted average COVID-19 incidence rate $I^{N}_{d,w}$ for district $d$ in week $w$ as:

2 $$I^{N}_{d,w}=\sum\limits_{j\notin d}^{J}\omega^{N}_{d,j}\,i_{j,w},$$

where $i_{j,w}$ is the disease incidence rate of district $j$ in week $w$. The intuition behind this measure is that disease dynamics in other districts are more relevant the closer districts are connected to the own district. Absent physical distancing or mobility restrictions, we expect $I^{N}_{d,w}$ to have a positive effect on generation of new cases in district $d$. That is, an increase in average—proximity weighted—incidence in other districts, will lead to higher case numbers in the home district.

Our final estimating dataset consists of 8,421 district $\times$ week level observations.^16^ Summary statistics of key variables are presented in Table 3.

## Empirical Strategy

The following OLS regression model forms the basis of our empirical analysis:

3 $$i_{d,w}=\gamma\,I^{N}_{d,w-1}+\beta\,i_{d,w-1}+\pi_{d}+\tau_{s(w)}+\varepsilon_{d,w}.$$

The number of new infections per inhabitant in district $d$ and week $w$ is represented by $i_{d,w}$. Our main regressor of interest is $I^{N}_{d,w-1}$, the network-proximity-weighted COVID-19 incidence rate in districts $j\neq d$ and week $w-1$. We allow for a one-week lag to take into account that the median serial interval—the time period between the onset of symptoms with the primary patient and the onset of symptoms with the secondary patient—is around 4–5 days (Nishiura et al 2020 and https://www.rki.de/DE/Content/InfAZ/N/Neuartiges_Coronavirus/Steckbrief.html).^17^^,^^18^ The coefficient $\gamma$ represents the network transmission intensity, i.e., the number of new cases per capita in the home district generated by an increase of network-proximity-weighted incidence rate of one.^19^

In addition to new cases generated via interaction with individuals from other districts, local incidence rates also depend on past incidence within the home district. Uncontrolled, higher case numbers yesterday imply more infected individuals today. To capture these within-district dynamics, we account for last week’s incidence rate in the home district, $i_{d,w-1}$.^20^ The coefficient $\beta$ is interpretable as internal growth rate and reflects how many new COVID-19 cases per capita are generated from one additional case per inhabitant.

All regressions control for district fixed effects $\pi_{d}$ which capture any time-invariant characteristics that influence the propagation of the disease within a given district. Possible aspects are population size and density, demographic composition and, importantly, the average amount of people flowing in and out of the district. The location dummies also account for the position of the district in the network (e.g, distance to initial hotspots). The variation exploited in our analysis is thus not generated by the structure of, or the position within, the network but by changes in network-proximity-weighted case numbers across all districts.^21^ General time-varying shocks symmetrically affecting all districts located within state $s$ are absorbed by state $\times$ week fixed effects $\tau_{s(w)}$. In particular, they capture the aggregate dynamics of the epidemic (depicted in Fig. 1a) as well as changes in state-wide testing capacities and strategies over time. They also account for potentially slightly differing policies and attitudes across states and time. Finally, the idiosyncratic error term—symbolised by $\varepsilon_{d,w}$—is clustered at the district level.

A natural conjecture is that disease transmission dynamics—within and between districts—change after implementation of COVID-19-related containment policies. To test whether this is the case, we estimate separate slope coefficients for the pre-policy period (weeks 1–11) and post-policy period (weeks 12–22).^22^ Extending regression model (3) to include an indicator $P$ for the weeks after which policies had been implemented, the estimating equation becomes:

4 $$\begin{aligned}[b]i_{d,w}&=\gamma_{1}\left(1-P\right)I^{N}_{d,w-1}+\gamma_{2}\,P\,I^{N}_{d,w-1}+\beta_{1}\left(1-P\right)i_{d,w-1}+\beta_{2}\,P\,i_{d,w-1}\\&\quad +\pi_{d}+\tau_{s(w)}+\varepsilon_{d,w}.\end{aligned}$$

Coefficient $\gamma_{1}$ captures the effect of the network-connectivity-weighted incidence before the implementation of policies, whereas $\gamma_{2}$ is the estimate for the post-policy period. Analogously, $\beta_{1}$ and $\beta_{2}$ captures within-district transmission dynamics before and after policies.

## Results

In this section, we first present the main results of our regression analysis. In the second part, we then turn to investigating potential mechanisms.

### Spatial networks and interdistrict disease transmission

As a benchmark, we start by estimating a version of regression equation (4) without network effects. That is, we regress current COVID-19 incidence rate on last week’s incidence rate. As outlined above, we estimate separate slope coefficients for the pre-and post-policy periods. Column (1) of Table 1 presents the results. The coefficient of 1.184 implies that a rise in the incidence rate of 1 this week, increases the incidence rate by 1.184 next week. Thus, even before accounting for cross-district infections, within-district transmission is intense enough to lead to sustained growth in case numbers. This is consistent with the rapid growth of new infections observed during the initial phases of the epidemic. After the implementation of containment policies in week 12, however, dynamics change significantly. The point estimate of 0.656 is now below one, implying—all else equal—a slow dissipation of the disease.

**Table 1:** Spatial networks and interdistrict disease transmission

		Incidence in week $w$			
		(1)	(2)	(3)	(4)
**Pre-policies**	Incidence in week $w-1$	1.184***	1.150***	1.149***	1.158***
		(0.347)	(0.341)	(0.340)	(0.338)
	Network-weighted		0.819***	0.850**	1.737***
	incidence in week $w-1$		(0.302)	(0.330)	(0.624)
**Post-policies**	Incidence in week $w-1$	0.656***	0.592***	0.591***	0.589***
		(0.033)	(0.061)	(0.063)	(0.062)
	Network-weighted		0.240**	0.248**	0.373**
	incidence in week $w-1$		(0.102)	(0.106)	(0.152)
	**Network**		**Mobility**	**Commuter**	**Facebook**
	District FE	Yes	Yes	Yes	Yes
	State $\times$ week FE	Yes	Yes	Yes	Yes
	R‑squared	0.827	0.831	0.831	0.831
	Observations	8,421	8,421	8,421	8,421
	BIC	−133,150	−133,344	−133,336	−133,372

Notes: This table reports estimates of Eq. 4 using the OLS estimator. Standard errors are clustered at the district level and reported in parentheses. Dependent variable is the weekly COVID-19 incidence rate in district $d$. ‘Incidence in week $w-1$’ represents the lagged weekly incidence rate in district $d$, and ‘Network-weighted incidence in week $w-1$’ is the lagged network-proximity-weighted weekly incidence rate (defined according to Eq. 1). ‘Pre-policies’ refers to weeks 1–11, ‘Post-policies’ to weeks 12–22. *$p<0.10$, **$p<0.05$, ***$p<0.01$.

In column (2), we add the mobility-network-weighted incidence rate to the list of regressors. Looking first at within-district transmission dynamics, the estimates remain very stable compared to column (1). Central to our analysis, we find that the structure of the spatial network predicts COVID-19 case numbers in the home district. The coefficient of 0.819 implies that if (proximity-weighted) average incidence within the mobility network increases by one, this generates 0.819 secondary cases per capita in the home district. The size of the point estimator is statistically indistinguishable from the within-district effect, illustrating the importance of taking into account network effects when trying to understand the spatiotemporal transmission of the COVID-19 epidemic.^23^ This is further illustrated by the difference in the Bayesian information criterion (BIC). Compared to column (1) the information content substantially increases when network effects are accounted for. Combined, the within and network transmission coefficients imply that one additional COVID-19 case per inhabitant in each region generates 1.97 ($1.15+0.819$) new cases per capita.

Paralleling within-district dynamics, network transmission intensity decreases after implementation of COVID-19-containment policies.^24^ Compared to the pre-policy period the point estimate for network transmission drops by 71%, whereas it is reduced by 49% for within-district transmission. There are multiple potential explanations for the particularly stark drop in cross-district transmission intensity. These will be discussed in the next section. First, however, we investigate the relevance of the remaining two types of networks in explaining inter-district disease transmission.

Column (3) tests whether the structure of the commuter network predicts the spatiotemporal spread of the COVID-19 epidemic. This is the case; the pattern of results is qualitatively equivalent to column (2). Furthermore, the size of point estimates as well as the information content—measured by the BIC—remain practically unaltered. This is unsurprising given the high degree of similarity in the structure of the commuter and mobility networks (see Table 4).

In the last column of Table 1, we investigate the ability of social networks, proxied by the SCI, to explain cross-district transmission dynamics. The point estimate for the social-network-proximity-weighted incidence rate of 1.737 is substantially larger than for the mobility and commuter networks. Combined, the network and within district transmission estimates imply that one COVID-19 case generates 2.89 new cases per capita. This suggests that social ties are a particularly relevant dimension along which diseases spread across space and time. A possible explanation is that while mobility and commuter networks capture movement of people, social networks (additionally) contain information on the quality of interaction. Social contact is likely to be more intimate than casual (or professional) interactions, thereby increasing the risk of infection. This additional information content is also reflected in the BIC, which is higher than for the mobility and commuter networks. This is a remarkable result. Even though, social network connectivity captures online friendship intensity and not necesarily physical contacts, the social network has higher predictive power than the mobility network. The latter in fact captures physical population movements. In analogy to previous results, the network effect drops markedly after the introduction of containment policies in week 12. In fact, the reduction in coefficient size of 79% is more pronounced than for the two other types of networks.^25^

In Table 6, we re-run regression model (4) and simultaneously account for multiple spatial networks, rather than just one. That is, we run a statistical horse race. The key insight from this exercise is that—while the estimates become more noisy—the size of the social-media-network-weighted incidence rates remains remarkably stable irrespective of which additional network is accounted for. Point-estimates of the other networks are not stable and are typically reduced by an order of magnitude when controlling for multiple networks. This is consistent with previous results and indicates that social network connectivity is—among the networks investigated here—the most important explanatory factor.

To sum up, Table 1 presents two main results. First, the structure of spatial networks—particularly social networks—strongly influences the spread of COVID-19 over space and time. The higher transmission intensity in well connected districts, the higher is future disease incidence in the home district. The existence of spillovers suggests the need for coordination of containment policies among well connected areas. Unilateral policies may prove (relatively) ineffective if transmission across districts is not stopped. For example, the localised easing of restrictions could have negative effects on disease containment in well connected regions. Second, network transmission intensity substantially drops after introduction of containment policies. Understanding the underlying causes is key in deriving policy implications. In the last section of this paper, we analyse potential mechanisms. First, however, we briefly discuss the validity and robustness of the results presented in Table 1.

One concern is that our OLS estimates are severely biased due to the dynamic regression setup. However, given the relatively large number of time periods included in our analysis, this is unlikely to be the case. To illustrate this formally, we re-run regressions of Table 1 using the difference GMM estimator developed in Arellano and Bond (1991). This estimator was specifically designed to produce consistent estimates in fixed effects models with lagged dependent variables. Reassuringly, the GMM estimation approach produces very similar results (Table 7). Similarly, we find that standard errors remain stable when we allow for spatial autocorrelation across districts using the Conley (1999) procedure or two-way cluster along the district and week dimension (Tables 8–9).^26^ Additionally, we show that the pattern of results remains unchanged if we look at absolute numbers of new cases rather than incidence rates (Table 10). Further, Table 11 illustrates that our results do not depend on week 12 as the cut-off point for the policy regimes. The results remain qualitatively unchanged if we move the cut-off to week 13, i.e. the week in which the federal government imposed nationwide restrictions. Finally, we show that the reduction in network transmission intensity is not driven by the period of severe restrictions (i.e. weeks 12–16). Estimating an additional separate slope coefficient for weeks 17–22 documents that, if anything, transmission intensity is even lower during this last period in our sample (Table 12).

Before investigating potential mechanisms underlying our main results, we want to highlight an important limitation of our study. The networks discussed here may not only facilitate the spread of COVID-19 infections, but also the spread of disease-related information. For example, increases in observed COVID-19 cases in the early stages of the pandemic could have been the result of higher testing capabilities or higher awareness of the disease itself rather than a consequence of actually increasing incidence rates. At the same time higher awareness about COVID-19 as well as information about testing possibilities can spread through social networks, leading to higher detection rates in neighbouring counties. In the presence of such effects, we would overestimate the effect of network connectivity on the diffusion of COVID-19 in the early stage of the pandemic. Unfortunately, the data structure does not allow us to disentangle these two processes.

### Structure, quantity and quality of interdistrict interaction

As documented above, the effect of network-proximity-weighted COVID-19 incidence drops substantially after the introduction of containment policies. Three non-exclusive mechanisms could underlie this result:

(i) Change in network structure: If the relative strength of bilateral links changes after introduction of containment policies—i.e. if the structure of the network changes—our predetermined networks would do a poor job at capturing actual cross-district interaction. As a consequence, the information contained in the network-proximity weighted average COVID-19 incidence rate would be very noisy after week 12. This, in turn, could bias estimates towards zero and explain the drop in size of the network transmission intensity coefficient.
(ii) Change in quantity: The level of inter-district movement drops substantially after the introduction of containment policies (cf. Fig. 1b). Therefore, the reduction in coefficient size could simply reflect the fact that fewer individuals move between districts. The district fixed effects in our regression setup control for differences in the size of average total inflows, but do not account for changes in quantities over time.
(iii) Change in quality: Qualitative aspects of inter-district movement and interaction could change. For example, adherence to hygiene and physical distancing guidelines, can reduce infection risks even if people move across space. Similarly, the curtailment of large social events is likely to have reduced the nature of contact (e.g., preventing super-spreader events) and thus the spatial disease transmission dynamics, even conditional on mobility.

To empirically analyse the relevance of these three potential mechanisms, we require time-varying information on interaction between districts. While unattainable for the commuter and social networks, data on interdistrict mobility are available at a daily interval for the whole sample period (i.e., weeks 1–22 of 2020). In the subsequent analysis, we therefore focus on the mobility network. We first use these observed mobility flows to investigate if the structure of the network changes over time. To this end, we compute the network proximity weights $\omega^{N}_{d,j}$ separately for each of the 22 weeks (cf. Eq. 1). As an initial step in gauging whether the structure of the mobility network changes, we look at the correlation between the weights computed from total mobility in 2019 and the weekly weights based on data from 2020. Over the 22-week period, the correlation between the time-invariant and the weekly updated weights is on average 0.997. Although there is a drop in correlation observable in week 12, this change is quantitatively small. Correlation drops from 0.996 in week 11 to 0.994 in week 12.^27^

The high correlation in bilateral connectivity over time strongly suggests that changes in the structure of networks is an unlikely explanation for the drop in the network transmission intensity observed after the implementation of containment policies. To formally confirm this impression, we use the time-varying weights to compute the network-proximity-weighted COVID-19 incidence rates according to Eq. 2. We then re-run the regression of column (2), Table 1. As expected, the estimates as well as information content (BIC)—both presented in Table 2 column (1)—remain virtually unaltered when using the updated weights to compute network exposure to COVID-19. Crucially, there is still a large and statistically significant reduction in network transmission intensity after the introduction of containment policies in week 12. This documents that changes in the structure of the network are not driving the decline in network transmission coefficient after introduction of containment policies.

The drop in the absolute amount of mobility after week 11 is the second plausible explanation for the reduction in network transmission intensity. While the results of column (1) illustrate that the decline was proportional across districts—i.e. the relative importance of links and therefore the network structure did not change—the reduction in absolute numbers varies across districts. For populous districts, the absolute drop in inflows is much larger than for small districts. These changes are not accounted for by the relative weights or the district fixed effects and hence could bias our estimates towards zero. To test if changes in the quantity of interdistrict movement explain the post-policy reduction in network transmission intensity, we first simply control for the total inflows into a district in a given week. As shown in column (2), the coefficients of network-transmission-weighted incidence remain very stable. Importantly, the magnitude of the drop in coefficient size after week 12 is unchanged. On the other hand, the point estimate of uninteracted mobility (i.e., total inflows into a given district) is extremely small and statistically non-significant.^28^^,^^29^ The same holds if we add total mobility to the regressions of Table 1 (see Table 5 for the results).

To further show that changes in levels of mobility do not drive the pattern of results, we take into account the absolute volume of population flows and compute network-proximity weighted incidence using absolute observed number of mobility as weights.^30^ Compared to column (1), the modification is thus that bilateral mobility weights are not normalised.^31^ The resulting point estimates imply that COVID-19 incidence rate in the home district increases by 0.618 with a one unit increase in absolute-weighted network case incidence in the pre-policy period, and by 0.09 in the post-policy period (column (3)). In relative terms, the size of reduction is similar to column (1), and if anything, stronger (72% vs 85%). These results illustrate that changes in the amount of inter-district movements cannot explain why we observe a reduction in transmission intensity across districts.

**Table 2:** Structure and quantity of mobility

		Incidence in week $w$		
		(1)	(2)	(3)
**Pre-policies**	Incidence in week $w-1$	1.146***	1.147***	1.170***
		(0.339)	(0.339)	(0.339)
	Updated network-weighted	0.857***	0.856***	0.618***
	incidence in week $w-1$	(0.301)	(0.300)	(0.199)
**Post-policies**	Incidence in week $w-1$	0.587***	0.587***	0.649***
		(0.060)	(0.060)	(0.037)
	Updated network-weighted	0.237**	0.236**	0.094
	incidence in week $w-1$	(0.092)	(0.092)	(0.059)
	Total Inflows $w-1$		0.000	
			(0.000)	
	Network	Mobility	Mobility	Mobility
	Network weights	Normalised	Normalised	Absolute
	District FE	Yes	Yes	Yes
	State $\times$ week FE	Yes	Yes	Yes
	R‑squared	0.831	0.831	0.827
	Observations	8421	8421	8421
	BIC	−133,363	−133,355	−133,171

Notes: This table reports estimates of Eq. 4 using the OLS estimator. Standard errors are clustered at the district level and reported in parentheses. Dependent variable is the weekly COVID-19 incidence rate in district $d$. ‘Incidence in week $w-1$’ represents the lagged weekly incidence rate in district $d$, and ‘Updated network-weighted incidence in week $w-1$’ is the lagged network-proximity-weighted weekly incidence rate (defined according to Eq. 1 and computed using observed weekly mobility data). ‘Total Inflows $w-1$’ is the total number of journeys to district $d$ that originate in other districts. ‘Pre-policies’ refers to weeks 1–11, ‘Post-policies’ to weeks 12–22. *$p<0.10$, **$p<0.05$, ***$p<0.01$.

Taken together, the results presented in Table 2 document that the decline in the size of network effect after the introduction of containment policies is neither due to changes in the structure of networks nor the drop in mobility levels. Though not empirically testable, the natural conjecture then is that qualitative aspects of mobility must have changed. A main policy implication thus is that changing qualitative aspects of mobility are far more important in slowing down the spread of the disease across space than the reduction of mobility itself. Due to the lack of data, we are not able to pinpoint which specific aspect of quality is particularly important in explaining the reduction in cross-regional transmission intensity. Possible aspects include (but are not restricted to): Changes in behaviour (e.g., adherence to physical distancing guidelines), changes in means of transports used, or changes in the type of trips (e.g., travelling in groups or individually). Identifying the relative contribution of these factors is an important question left for future research.

## Conclusion

This paper illustrates that spatial networks are important in understanding the spread of COVID-19 over space and time. This is particularly true during early stages of the epidemic, when no containment policies are in place. After the introduction of such policies, the effect of networks on cross-regional transmission drops markedly. Our results suggest that this reduction is primarily due to a change in quality—not quantity—of interregional movements. An important policy implication of this finding is that changing qualitative aspects of mobility are far more important in slowing down the spread of the disease across space than the reduction of mobility itself. When interpreting our results, it is important to keep in mind that these results could be specific to Germany, where legitimacy of the government—and therefore degree of adherence to guidelines—is high.

## Figures and Tables

**Fig. 4:**
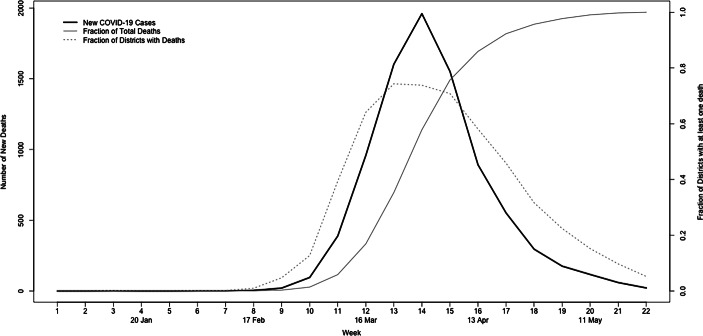
Figure depicts new weekly COVID-19-related deaths. (Data version: 9 June 2020)

**Fig. 5:**
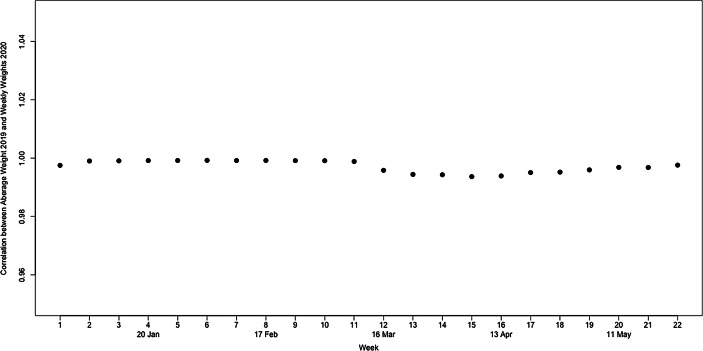
Correlation coefficients between time-invariant network proximty weights (average 2019) and time-varying network proximity weights (based on weekly mobility data)

## Appendix

### Supporting information

**Table 3:** Descriptive Statistics key variables

**Variable**	**Mean**	**Std. Dev.**	**Min.**	**Max.**	**Obs.**
	Overall				
Incidence	0.00011	0.00021	0	0.00422	8421
Mobility-network-proximity-weighted incidence	0.00011	0.00017	0	0.00206	8421
Commuter-network-proximity-weighted incidence	0.00011	0.00017	0	0.00196	8421
Social-network-proximity-weighted incidence	0.00010	0.00016	0	0.00164	8421

**Table 4:** Correlations District-Pair-Level Network Proximity Measures

	Mobility Network	Commuting Network	Social Network
Mobility Network	1		
Commuting Network	0.972	1	
Social Network	0.864	0.846	1

Notes: Table depicts the correlation between bilateral connectivity within the mobility, commuting and social network. Connectivity is computed according to Eq. 1.

**Table 5:** Spatial networks and interdistrict disease transmission

		Incidence in week $w$			
		(1)	(2)	(3)	(4)
**Pre-policies**	Incidence in week $w-1$	1.185***	1.151***	1.150***	1.159***
		(0.347)	(0.341)	(0.340)	(0.338)
	Network-weighted		0.818***	0.849**	1.735***
	incidence in week $w-1$		(0.302)	(0.329)	(0.623)
**Post-policies**	Incidence in week $w-1$	0.656***	0.591***	0.591***	0.589***
		(0.033)	(0.061)	(0.063)	(0.062)
	Network-weighted		0.240**	0.248**	0.372**
	incidence in week $w-1$		(0.102)	(0.106)	(0.152)
	Total Inflows $w-1$		0.240**	0.248**	0.372**
			(0.102)	(0.106)	(0.152)
	**Network**		**Mobility**	**Commuter**	**Facebook**
	District FE	Yes	Yes	Yes	Yes
	State $\times$ week FE	Yes	Yes	Yes	Yes
	R‑squared	0.827	0.831	0.831	0.831
	Observations	8,421	8,421	8,421	8,421
	BIC	−133,150	−133,344	−133,336	−133,372

Notes: This table reports estimates of Eq. 4 using the OLS estimator. Standard errors are clustered at the district level and reported in parentheses. Dependent variable is the weekly COVID-19 incidence rate in district $d$. ‘Incidence in week $w-1$’ represents the lagged weekly incidence rate in district $d$, and ‘Network-weighted incidence in week $w-1$’ is the lagged network-proximity-weighted weekly incidence rate (defined according to Eq. 1). ‘Total Inflows $w-1$’ is the total number of journeys to district $d$ that originate in other districts. ‘Pre-policies’ refers to weeks 1–11, ‘Post-policies’ to weeks 12–22. *$p<0.10$, **$p<0.05$, ***$p<0.01$.

**Table 6:** Multiple Networks: Spatial networks and interdistrict disease transmission

		Incidence in week $w$			
		(1)	(2)	(3)	(4)
**Pre-policies**	Incidence in week $w-1$	1.150***	1.167***	1.164***	1.167***
		(0.341)	(0.340)	(0.342)	(0.341)
	Mobility-weighted	0.397	−0.372		−0.556
	incidence in week $w-1$	(1.742)	(0.675)		(1.831)
	Commuter-weighted	0.442		−0.288	0.194
	incidence in week $w-1$	(1.857)		(0.585)	(1.661)
	Facebook-weighted		2.437	2.253*	2.426
	incidence in week $w-1$		(1.533)	(1.337)	(1.513)
**Post-policies**	Incidence in week $w-1$	0.591***	0.587***	0.588***	0.588***
		(0.062)	(0.063)	(0.063)	(0.063)
	Mobility-weighted	0.258	0.059		0.207
	incidence in week $w-1$	(0.180)	(0.087)		(0.180)
	Commuter-weighted	−0.020		0.021	−0.184
	incidence in week $w-1$	(0.181)		(0.117)	(0.249)
	Facebook-weighted		0.296*	0.347*	0.332*
	incidence in week $w-1$		(0.168)	(0.202)	(0.200)
	District FE	Yes	Yes	Yes	Yes
	State $\times$ week FE	Yes	Yes	Yes	Yes
	R‑squared	0.831	0.831	0.831	0.832
	Observations	8,421	8,421	8,421	8,421
	BIC	−133,326	−133,356	−133,354	−133,342

Notes: This table reports estimates of Eq. 4 using the OLS estimator. Standard errors are clustered at the district level and reported in parentheses. Dependent variable is the weekly COVID-19 incidence rate in district $d$. ‘Incidence in week $w-1$’ represents the lagged weekly incidence rate in district $d$, and the different ‘Network-weighted incidence in week $w-1$’ variables are the lagged network-proximity-weighted weekly incidence rate (defined according to Eq. 1). ‘Pre-policies’ refers to weeks 1–11, ‘Post-policies’ to weeks 12–22. *$p<0.10$, **$p<0.05$, ***$p<0.01$.

### Robustness

**Table 7:** Arellano and Bond (1991) difference GMM estimator: Spatial networks and interdistrict disease transmission

		Incidence in week $w$			
		(1)	(2)	(3)	(4)
**Pre-policies**	Incidence in week $w-1$	1.279***	1.238***	1.097***	1.242***
		(0.357)	(0.351)	(0.256)	(0.347)
	Network-weighted		0.901***	0.899***	1.914***
	incidence in week $w-1$		(0.323)	(0.327)	(0.662)
**Post-policies**	Incidence in week $w-1$	0.776***	0.702***	0.754***	0.696***
		(0.040)	(0.074)	(0.090)	(0.076)
	Network-weighted		0.245**	0.326***	0.386**
	incidence in week $w-1$		(0.111)	(0.083)	(0.165)
	**Network**		**Mobility**	**Commuter**	**Facebook**
	District FE	Yes	Yes	Yes	Yes
	State $\times$ week FE	Yes	Yes	Yes	Yes
	Observations	8,421	8,421	8,421	8,421

Notes: This table reports estimates of Eq. 4 using the OLS estimator. Standard errors are clustered at the district level and reported in parentheses. Dependent variable is the weekly COVID-19 incidence rate in district $d$. ‘Incidence in week $w-1$’ represents the lagged weekly incidence rate in district $d$, and ‘Network-weighted incidence in week $w-1$’ is the lagged network-proximity-weighted weekly incidence rate (defined according to Eq. 1). ‘Pre-policies’ refers to weeks 1–11, ‘Post-policies’ to weeks 12–22. *$p<0.10$, **$p<0.05$, ***$p<0.01$.

**Table 8:** Conley standard errors: Spatial networks and disease transmission

		Incidence in week $w$			
		(1)	(2)	(3)	(4)
**Pre-policies**	Incidence in week $w-1$	1.184***	1.150***	1.149***	1.158***
		[0.345]	[0.330]	[0.329]	[0.330]
	Network-weighted		0.819***	0.850**	1.737***
	incidence in week $w-1$		[0.290]	[0.307]	[0.619]
**Post-policies**	Incidence in week $w-1$	0.656***	0.592***	0.591***	0.589***
		[0.039]	[0.045]	[0.046]	[0.042]
	Network-weighted		0.240***	0.248***	0.373***
	incidence in week $w-1$		[0.049]	[0.052]	[0.074]
	**Network**		**Mobility**	**Commuter**	**Facebook**
	District FE	Yes	Yes	Yes	Yes
	State $\times$ week FE	Yes	Yes	Yes	Yes
	R‑squared	0.827	0.831	0.831	0.831
	Observations	8,421	8,421	8,421	8,421
	BIC	−133,150	−133,344	−133,336	−133,372

Notes: This table reports estimates of Eq. 4 using the OLS estimator. Standard errors computed using the approach of Conley (1999) reported in brackets (cut-off 2 degrees) at the district level and reported in brackets. Dependent variable is the weekly COVID-19 incidence rate in district $d$. ‘Incidence in week $w-1$’ represents the lagged weekly incidence rate in district $d$, and ‘Network-weighted incidence in week $w-1$’ is the lagged network-proximity-weighted weekly incidence rate (defined according to Eq. 1). ‘Pre-policies’ refers to weeks 1–11, ‘Post-policies’ to weeks 12–22. *$p<0.10$, **$p<0.05$, ***$p<0.01$.

**Table 9:** Two-way clustering: Spatial networks and interdistrict disease transmission

		Incidence in week $w$			
		(1)	(2)	(3)	(4)
**Pre-policies**	Incidence in week $w-1$	1.184***	1.150***	1.149***	1.158***
		(0.361)	(0.338)	(0.337)	(0.345)
	Network-weighted		0.819**	0.850**	1.737**
	incidence in week $w-1$		(0.336)	(0.372)	(0.711)
**Post-policies**	Incidence in week $w-1$	0.656***	0.592***	0.591***	0.589***
		(0.059)	(0.085)	(0.086)	(0.088)
	Network-weighted		0.240**	0.248**	0.373**
	incidence in week $w-1$		(0.098)	(0.104)	(0.147)
	**Network**		**Mobility**	**Commuter**	**Facebook**
	District FE	Yes	Yes	Yes	Yes
	State $\times$ week FE	Yes	Yes	Yes	Yes
	R‑squared	0.827	0.831	0.831	0.831
	Observations	8,421	8,421	8,421	8,421
	BIC	−133,150	−133,344	−133,336	−133,372

Notes: This table reports estimates of Eq. 4 using the OLS estimator. Standard errors are two-way clustered along the district and week dimension and reported in parentheses. Dependent variable is the weekly COVID-19 incidence rate in district $d$. ‘Incidence in week $w-1$’ represents the lagged weekly incidence rate in district $d$, and ‘Network-weighted incidence in week $w-1$’ is the lagged network-proximity-weighted weekly incidence rate (defined according to Eq. 1). ‘Pre-policies’ refers to weeks 1–11, ‘Post-policies’ to weeks 12–22. *$p<0.10$, **$p<0.05$, ***$p<0.01$.

**Table 10:** Absolute case numbers: Spatial networks and disease transmission

		Cases in week $w$			
		(1)	(2)	(3)	(4)
**Pre-policies**	Cases in week $w-1$	1.564**	1.527**	1.521**	1.509**
		(0.676)	(0.671)	(0.667)	(0.657)
	Network-weighted		0.348***	0.373***	2.064***
	cases in week $w-1$		(0.128)	(0.126)	(0.787)
**Post-policies**	Cases in week $w-1$	0.750***	0.743***	0.744***	0.742***
		(0.036)	(0.040)	(0.039)	(0.039)
	Network-weighted		0.033***	0.029***	0.172***
	cases in week $w-1$		(0.012)	(0.011)	(0.058)
	**Network**		**Mobility**	**Commuter**	**Facebook**
	District FE	Yes	Yes	Yes	Yes
	State $\times$ week FE	Yes	Yes	Yes	Yes
	R‑squared	0.827	0.831	0.831	0.831
	Observations	8,421	8,421	8,421	8,421
	BIC	73,100	73,063	73,059	73,038

Notes: This table reports estimates of Eq. 4 using the OLS estimator. Standard errors are clustered at the district level and reported in parentheses. Dependent variable is the weekly number of new COVID-19 cases in district $d$. ‘Cases in week $w-1$’ represents the lagged weekly new cases in district $d$, and ‘Network-weighted cases in week $w-1$’ is the lagged network-proximity-weighted weekly case number (defined according to Eq. 1). ‘Pre-policies’ refers to weeks 1–11, ‘Post-policies’ to weeks 12–22. *$p<0.10$, **$p<0.05$, ***$p<0.01$.

**Table 11:** Week 13 as beginning of post-policy period: Spatial networks and disease transmission

		Incidence in week $w$			
		(1)	(2)	(3)	(4)
**Pre-policies**	Incidence in week $w-1$	1.321***	1.241***	1.233***	1.238***
		(0.183)	(0.208)	(0.210)	(0.203)
	Network-weighted		0.591***	0.668***	1.126***
	incidence in week $w-1$		(0.212)	(0.223)	(0.328)
**Post-policies**	Incidence in week $w-1$	0.590***	0.514***	0.516***	0.511***
		(0.020)	(0.035)	(0.036)	(0.038)
	Network-weighted		0.268***	0.274***	0.420***
	incidence in week $w-1$		(0.074)	(0.075)	(0.118)
	**Network**		**Mobility**	**Commuter**	**Facebook**
	District FE	Yes	Yes	Yes	Yes
	State $\times$ week FE	Yes	Yes	Yes	Yes
	R‑squared	0.844	0.851	0.851	0.852
	Observations	8,421	8,421	8,421	8,421
	BIC	-134,049	-134,397	-134,399	-134,480

Notes: This table reports estimates of Eq. 4 using the OLS estimator. Standard errors are clustered at the district level and reported in parentheses. Dependent variable is the weekly COVID-19 incidence rate in district $d$. ‘Incidence in week $w-1$’ represents the lagged weekly incidence rate in district $d$, and ‘Network-weighted incidence in week $w-1$’ is the lagged network-proximity-weighted weekly incidence rate (defined according to Eq. 1). ‘Pre-policies’ refers to weeks 1–12, ‘Post-policies’ to weeks 13–22. *$p<0.10$, **$p<0.05$, ***$p<0.01$.

**Table 12:** Three time periods: Spatial networks and disease transmission

		Incidence in week $w$			
		(1)	(2)	(3)	(4)
**Pre-policies**	Incidence in week $w-1$	1.240***	1.205***	1.206***	1.220***
		(0.342)	(0.332)	(0.332)	(0.330)
	Network-weighted		0.812***	0.830***	1.738***
	incidence in week $w-1$		(0.297)	(0.319)	(0.617)
**Post-policies**	Incidence in week $w-1$	0.764***	0.692***	0.692***	0.689***
		(0.025)	(0.056)	(0.057)	(0.055)
	Network-weighted		0.225**	0.232**	0.349***
	incidence in week $w-1$		(0.089)	(0.092)	(0.126)
**First-relaxations**	Incidence in week $w-1$	0.579***	0.527***	0.534***	0.535***
		(0.031)	(0.033)	(0.034)	(0.035)
	Network-weighted		0.202***	0.189***	0.249***
	incidence in week $w-1$		(0.039)	(0.040)	(0.058)
	**Network**		**Mobility**	**Commuter**	**Facebook**
	District FE	Yes	Yes	Yes	Yes
	State $\times$ week FE	Yes	Yes	Yes	Yes
	R‑squared	0.820	0.825	0.825	0.826
	Observations	8,421	8,421	8,421	8,421
	BIC	-132,847	-133,046	-133,032	-133,070

Notes: This table reports estimates of Eq. 4 using the OLS estimator. Standard errors are clustered at the district level and reported in parentheses. Dependent variable is the weekly COVID-19 incidence rate in district $d$. ‘Incidence in week $w-1$’ represents the lagged weekly incidence rate in district $d$, and ‘Network-weighted incidence in week $w-1$’ is the lagged network-proximity-weighted weekly incidence rate (defined according to Eq. 1). ‘Pre-policies’ refers to weeks 1–11, ‘Post-policies’ to weeks 12–26, and ‘First-relaxations’ to weeks 17–22. *$p<0.10$, **$p<0.05$, ***$p<0.01$.

## Footnotes

1 See, for example, https://coronavirus.jhu.edu/map.html.

2 See Sect. 4 for more details on case data.

3 The spatiotemporal dynamics of COVID-19-related deaths exhibit a similar pattern, although shifted by 2–3 weeks. This can be explained by the lag between infection and death (Verity et al 2020). See Fig. 4.

4 Implementing health policies falls within the responsibility of the states (‘Bundesländer’). As such, there is some (minor) variation in the type and timing of containment policies. Such differences are accounted for in our estimation approach (see Sect. 5).

5 For example, smaller shops with sales areas up to 800 square metres were allowed to re-open on 20 April 2020.

6 Qualitatively, all subsequently presented results remain unchanged if we additionally split the post-policy period into two subgroups: ‘full restrictions’ (weeks 12–16), and ‘relaxed restrictions (weeks 17–22).

7 The data are available at https://bit.ly/2YQlOo1. Districts are the third level of administrative division, thus corresponding to districts in England or counties in the US.

8 The focus on the first 22 weeks of 2020 is dictated by the availability of the mobile-phone-based mobility data (see below for more information). As of writing, these are only available for weeks 1–22.

9 Differences in the latter are accounted for in our estimation setup through inclusion of district fixed effects (see details below).

10 The data are commercially available at www.teralytics.de.

11 A trip is defined as any movement between two phone tower located in different district with a minimum of 60 minutes stationary time at one location.

12 The data are available here: https://bit.ly/2YLlVRK.

13 Information on how to gain access to the SCI are provided here: https://bit.ly/316FdCg.

14 If the SCI increases by a factor 2, doubles the probability that a Facebook user in district $j$ is connected to a Facebook user in district $j$.

15 Similarly, the correlation between the commuting and the social network is 0.85.

16 The ‘raw’ dataset consists of $401\times 22=8{,}822$ observations. However, because our estimating equation includes lagged values, the number of observations drops to $8{,}822-401=8{,}421$. See Sect. 5 below for more details.

17 The median incubation period, i.e., the time from the moment of exposure to the virus until symptoms of the disease appear, is estimated to be around 5–6 days (Nishiura et al 2020 and https://www.rki.de/DE/Content/InfAZ/N/Neuartiges_Coronavirus/Steckbrief.html). Consistent with a substantial proportion of pre-symptomatic transmission, this is longer than the estimated median serial interval.

18 Using lagged rather contemporary values has the additional advantage of avoiding issues of reverse causality and simultaneity, i.e., infections in partner districts being a function of disease incidence in the home district. More generally, using panel data insulates from the reflection problem (multidirectional causality) present in cross-section network analysis (see Manski (1993) and García-Jimeno et al (2022))

19 A potential concern with dynamic panel data models is that estimates are affected by the Nickell bias (Nickell 1981). However, due to the long time dimension and the high frequency of our data, the bias is of limited relevance in the context of our study.

20 The inclusion of a one week lag is motivated by studies that find highest viral loads at the time of symptom onset (i.e. 4–5 days after infection) and infectiousness to decline quickly within 7 days thereafter (He et al 2020).

21 As mentioned earlier, a concern is that the structure of the networks change. We address this issue in Sect. 6 below.

22 We have opted to estimate separate slope coefficients rather than introducing a simple interaction term. Econometrically, the two approaches are equivalent. However, differences in effect sizes can be more readily discernible when estimating separate slope coefficients.

23 The p‑value for coefficient equality is 0.549.

24 Combined, the network and within transmission are now below the critical value of one ($0.592+0.240=0.832$).

25 It is important to note that we do not claim to be able to isolate—i.e. causally separate—the effects of a specific network. The reason being the likely presence of latent networks. For the social media network, for example, it is plausible that friendships (i.e. connectivity) do not emerge randomly, but rather latent networks give rise to friendship connections across space (e.g., core-periphery spatial structures etc.). Consequently, social network connectivity might be associated with—and capture—these deeper, more persistent, network structures.

26 For the Conley (1999) standard errors, we report results where we allow for spatial autocorrelation across districts that declines linearly in distance up to a cut-off of 200 kilometres (representing the average diameter of a state). We have experimented with cut-offs ranging from 100 to 1,000 kilometres. The standard errors change very little (results available upon request).

27 Figure 5 in Appendix visualises the stability of the structure of the network over time.

28 The size of the point estimate implies that there is one additional COVID-19 case per capita for each one hundred billion (100,000,000,000) trips that started outside the district and ended in the home district.

29 The results remain unchanged if we allow the effect of mobility to differ between the pre- and post-policy period (i.e. if we estimate separate slope coefficients).

30 More specifically, we use absolute cross-district movements measured in millions.

31 Formally, the absolute weights are defined as $\omega^{N}_{d,j}=f_{d,j}$, where $f_{d,j}$ is the number of trips into district $d$ that originate from district $j$ in a given week.
